# Androgen and oestrogen receptor co-expression determines the efficacy of hormone receptor-mediated radiosensitisation in breast cancer

**DOI:** 10.1038/s41416-022-01849-9

**Published:** 2022-05-26

**Authors:** Anna R. Michmerhuizen, Lynn M. Lerner, Connor Ward, Andrea M. Pesch, Amanda Zhang, Rachel Schwartz, Kari Wilder-Romans, Joel R. Eisner, James M. Rae, Lori J. Pierce, Corey W. Speers

**Affiliations:** 1 Department of Radiation Oncology, University of Michigan, Ann Arbor, MI USA; 2 Rogel Cancer Center, University of Michigan, Ann Arbor, MI USA; 3 Program in Cellular and Molecular Biology, University of Michigan, Ann Arbor, MI USA; 4 Department of Pharmacology, University of Michigan, Ann Arbor, MI USA; 5 Innocrin Pharmaceuticals, Durham, NC USA; 6 Department of Internal Medicine, University of Michigan, Ann Arbor, MI USA

**Keywords:** Breast cancer, Breast cancer, Translational research, Radiotherapy, Hormonal therapies

## Abstract

**Purpose:**

Radiation therapy (RT) and hormone receptor (HR) inhibition are used for the treatment of HR-positive breast cancers; however, little is known about the interaction of the androgen receptor (AR) and estrogen receptor (ER) in response to RT in AR-positive, ER-positive (AR+/ER+) breast cancers. Here we assessed radiosensitisation of AR+/ER+ cell lines using pharmacologic or genetic inhibition/degradation of AR and/or ER.

**Methods:**

Radiosensitisation was assessed with AR antagonists (enzalutamide, apalutamide, darolutamide, seviteronel, ARD-61), ER antagonists (tamoxifen, fulvestrant) or using knockout of *AR*.

**Results:**

Treatment with AR antagonists or ER antagonists in combination with RT did not result in radiosensitisation changes (radiation enhancement ratios [rER]: 0.76–1.21). Fulvestrant treatment provided significant radiosensitisation of CAMA-1 and BT-474 cells (rER: 1.06–2.0) but not ZR-75-1 cells (rER: 0.9–1.11). Combining tamoxifen with enzalutamide did not alter radiosensitivity using a 1 h or 1-week pretreatment (rER: 0.95–1.14). Radiosensitivity was unchanged in *AR* knockout compared to Cas9 cells (rER: 1.07 ± 0.11), and no additional radiosensitisation was achieved with tamoxifen or fulvestrant compared to Cas9 cells (rER: 0.84–1.19).

**Conclusion:**

While radiosensitising in AR + TNBC, AR inhibition does not modulate radiation sensitivity in AR+/ER+ breast cancer. The efficacy of ER antagonists in combination with RT may also be dependent on AR expression.

## Graphical abstract

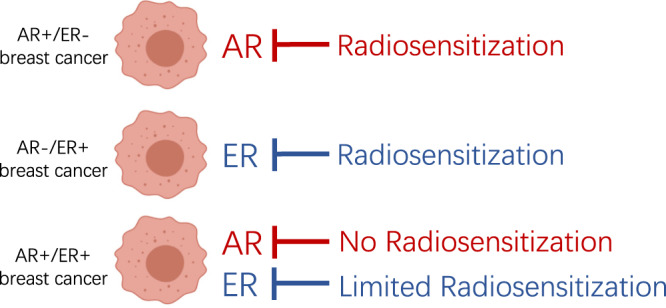

## Introduction

Breast cancer is a heterogeneous disease, classified largely by the expression of the estrogen receptor (ER), progesterone receptor (PR), and by amplification of the human epidermal growth factor receptor 2 (HER2) [1]. Over 80% of breast cancer patients have ER-positive (ER+) tumours that express ER [2], and the androgen receptor (AR) is co-expressed with the estrogen receptor in 70–95% of all ER+ breast cancers [3, 4]. AR expression is also found in 30–60% of non-ER expressing (ER-negative) tumours [5, 6] where the role of AR in radiosensitisation has previously been described [7, 8]. Functionally, both AR and ER have very similar roles as both are activated by hormone binding and act as transcription factors, binding to response elements in the nucleus [9]. AR has been shown to compete with ER for binding to estrogen response elements (EREs) therefore acting as an antagonist to ER signaling in AR+/ER+ breast cancers [10]. AR-targeting therapies have been investigated for their potential therapeutic use in the treatment of AR+ cancers. Multiple anti-androgen therapies have been developed first for use in patients with prostate cancer [11]. The potential therapeutic use of these agents, including second-generation anti-androgens apalutamide (ARN-509) [12], darolutamide (ODM-201) [13] and enzalutamide (MDV3100) [14], has been explored in other AR+ cancers, including AR+ breast cancers. Enzalutamide is the most widely used AR inhibitor and is currently being explored in multiple clinical trials in breast cancers (NCT04142060, NCT03207529, NCT02689427) [15]. Similarly, darolutamide (NCT03383679) and seviteronel (NCT04947189) are also currently being explored in AR+ breast cancers. Previous work by our group and others has demonstrated that AR inhibition is an effective strategy for radiosensitisation of triple-negative breast cancers (TNBCs) [7, 8, 16]. More recently, the role of the androgen receptor as a tumour suppressor in AR+/ER+ breast tumours in vitro and in vivo has been described, suggesting that AR activation, not inhibition, may provide a therapeutic benefit [17], and this is currently being tested in ongoing clinical trials using AR agonists with encouraging findings (NCT01616758, NCT02463032). Thus, seemingly conflicting data support both AR antagonism and agonism as treatment strategies in AR+ breast cancer suggesting a context-dependent effect that is currently poorly understood. While ongoing work seeks to understand how these receptors may be influencing tumorigenesis, there is still a clinical need for the development of better biomarkers of response for AR-targeted therapies, including the use of both agonists and antagonists. In addition, although AR and ER belong to the same class of steroid hormone nuclear transcription factors [9], the overlapping functions of these hormone receptors (HR) in treatment response have not been well characterised.

Further, the roles of both AR and ER in the radiation response are not well understood. Previous studies indicate that TNBC patients with low AR expression have decreased rates of local recurrence following radiation therapy (RT) and that AR protein expression is a biomarker of response in AR+ TNBC models [8]. Additional work has demonstrated that AR inhibition with the second-generation anti-androgen, enzalutamide, AR knockdown by siRNA, or treatment with the experimental dual AR and CYP17 lyase inhibitor, seviteronel, sensitises AR+ TNBC cells to radiation therapy [7, 8]. Further, our group and others have shown that inhibition or degradation of ER with tamoxifen or fulvestrant, respectively, is sufficient to radiosensitise ER+ breast cancer models [18–22]. Together these findings suggest a role for nuclear hormone receptors in the radiation response and warrant further investigation of AR and ER inhibition in AR+/ER+ breast cancer models.

Androgen receptor expression has also been investigated as a contributor to the radiation response in multiple types of cancer, including prostate cancer, breast cancer and glioblastoma [8–12]. AR has been shown to be a mediator of radioresistance in prostate cancer [23], and inhibition of AR with apalutamide results in an increased radiosensitivity through a decrease in non-homologous end-joining (NHEJ) efficiency [24, 25]. Additionally, in AR+ glioblastoma cell lines and xenograft models, inhibition of AR with enzalutamide or seviteronel has been shown to provide sensitisation to radiation therapy [26, 27]. These findings suggest that AR inhibition may be a generalisable strategy for the radiosensitisation of cancers with high AR expression. Previous studies, however, do not address the role of AR when expressed in combination with other hormone receptors, including the estrogen receptor, and context-dependent biological effects have yet to be described.

Having previously demonstrated that ER inhibition is an effective radiosensitisation strategy in ER+, AR-low breast cancers [22] and AR inhibition is a potentially effective radiosensitisation strategy in AR+/ER− breast cancer [8], the purpose of this study was to assess the effectiveness of AR inhibition as a radiosensitisation strategy in AR+/ER+ breast cancer models. In contrast to findings in other AR + diseases, including AR + TNBC, here we demonstrate that AR inhibition with enzalutamide, apalutamide, darolutamide and seviteronel, degradation of AR with the AR PROTAC degrader, ARD-61, or knockout of *AR* using CRISPR-Cas9, does not radiosensitise AR+/ER+ breast cancer cells in vitro. Additionally, dual inhibition or degradation of AR and ER is not sufficient, in most models, to radiosensitise AR+/ER+ breast cancer cells in vitro suggesting that these receptors may not be compensating for each other when co-expressed. Further, combination treatment of AR inhibition or knockout with ER inhibition or degradation does not have a synergistic effect on radiosensitisation, suggesting that AR and ER are not directly and exclusively compensating for their role in the radiation response. While our previous data suggest that inhibition or degradation of ER results in radiosensitisation [22], here we demonstrate that ER inhibition is not uniformly sufficient to radiosensitise AR+/ER+ breast cancer models. Therefore, the prior observed radiosensitisation was associated with AR-low/ER+ cell lines, further promoting the notion of an interaction between AR and ER in response to RT. Altogether these findings indicate a novel role for AR and ER signaling in AR+/ER+ breast cancer compared to the role of AR in TNBC or ER in AR-low/ER+ breast cancer models in response to radiation treatment with important clinical implications for this subset of patients.

## Materials and methods

### Cell culture

All cells were grown at 37 °C in a 5% CO_2_ incubator. MCF-7 cells were grown in DMEM media (ThermoFisher 11965092) containing 1% penicillin/streptomycin (ThermoFisher 15070063) and 10% fetal bovine serum (FBS; Atlanta Biologicals S11550H). BT-474 and ZR-75-1 cells were grown in RPMI 1640 media (ThermoFisher 11875093) containing 1% penicillin/streptomycin and 10% FBS. CAMA-1 cells were grown in EMEM media (ThermoFisher 15070063) containing 1% penicillin/streptomycin and 10% FBS. ACC-422 cells were grown in MEM media (ThermoFisher 11095080) containing 1X Insulin-Transferrin-Selenium-Ethanolamine (ITS-X; Gibco 51500056), 1% penicillin/streptomycin and 15% FBS. Where noted, charcoal stripped serum (CSS, Atlanta Biologicals S11650H) was used in place of FBS to remove hormones and growth factors. When CSS was used, the base media was also free of phenol-red. DNA fingerprinting was performed using short tandem repeat (STR) profiling at the University of Michigan Advanced Genomics Core. The MycoAlert Mycoplasma Detection kit (Lonza LT07) was used to test cells routinely for mycoplasma.

### Gene expression knockout

CRISPR cell lines were generated using the lentiCRISPRv2 plasmid (Addgene #98291) and the AR guide sequence (5’ CACCTCCAGCTTGATGCGAGCGTG 3’). As previously described [28], the lentiCRISPRv2 plasmid was digested with BsmB1 for 15 min at 55 degrees and gel-purified using the QIAquick Gel Extraction Kit (Qiagen #28706X4). Oligonucleotides were obtained from Integrated DNA Technologies and annealed at 95 degrees then cooled at 5 degrees/min. The guide sequences were ligated into the CRISPR plasmid and transformed into Stbl3 bacteria. Lentivirus was prepared using HEK-293T cells transfected with 1.5 μg PAX2 (Addgene #12260), 0.3 μg MD2g (Addgene #12259) and 1.5 μg plasmid in Opti-MEM media. DMEM media containing 30% FBS was used to produce the virus and media containing the virus was collected at 24 and 48 h post-transfection. Virus-containing media was spun down and cleared through a 0.45 micron filter before adding to cells with 0.8 μg/mL polybrene. The selection was performed with hygromycin (CAMA-1 or ZR-75-1: 500 μg/mL). CRISPR pools were used for all assays. Cas9 CRISPR control cells were made with a control guide targeting *AAVS1* (5’ CACCGGGGGCCACTAGGGACAGGAT 3’).

### Proliferation assays

Cells were plated in a 96-well plate and allowed to fix overnight. Cells were treated with media containing 1.0 nM–10 μM ARD-61. After growing for 72 h cell viability was assessed with AlamarBlue (ThermoFisher DAL1100) at a concentration of 10% of the well volume. Viability was calculated using a plate reader by measuring the absorbance of each well with an excitation wavelength of 540 nm and an emission wavelength of 590 nm. GraphPad Prism 8.0 was used to calculate a dose-response curve and half-maximal inhibitor concentration (IC50). Six technical replicates were used for each experiment, and the experiment was repeated three times (*n* = 3).

### Clonogenic survival assays

Cells were plated and allowed to fix overnight. Pretreatment (1–24 h) with drug-containing media was followed by radiation (0–6 Gy). After growth for 1–4 weeks, colonies were fixed with methanol/acetic acid and stained with crystal violet. Colonies with >50 cells were counted, and data were analysed using the linear-quadratic model. All combination groups were normalised to the drug-only control (treated with 0 Gy RT). GraphPad Prism 8.0 was used to visualise the data. All experiments used three technical replicates and were repeated in triplicate (*n* = 3).

### Western blotting

Cells were harvested at indicated time points. RIPA buffer (Thermo Fisher 89901) containing protease and phosphatase inhibitors (Sigma–Aldrich PHOSS-RO, CO-RO) was used to lyse cells. For nuclear fractionation experiments, the NE-PER Nuclear and Cytoplasmic Extraction Reagents (Thermo Scientific 78835) were used to separate the nuclear and cytoplasmic cellular fractions. All samples were sonicated and then standardised using a BCA assay. Samples were run in a 4–12% NUPAGE Bis-Tris protein gel at 120 V then transferred to a PVDF membrane, blocked in 5% milk in TBST, and primary antibody (AR: 1:1000, Millipore PG-21; ERα: 1:1000, Cell Signaling 8644; β-actin: 1:50,000, Cell Signaling 12262 S; LaminB1: 1:1000, Cell Signaling 12586; GAPDH: 1:1000, Cell Signaling 2118L) was added overnight. Anti-rabbit secondary antibody (1:10,000, Cell Signaling 7074S) was then added and blots were visualised using ECL Prime on a ChemiDoc Imaging System. ImageJ was used for the quantification of western blots.

### Reverse transcription and qPCR

To assess changes in mRNA levels, cells were first plated in complete media containing FBS. The next day, cells were pretreated with media without phenol-red, containing CSS for 48 h. Stimulation was then performed with β-estradiol or metribolone (R1881). After 24 h of stimulation, RNA was extracted with TRIzol using the miRNeasy kit for RNA isolation (Qiagen 217004). Reverse transcription was performed using random primers (Thermo Fisher 48190011), dNTPs (Thermo Fisher 18427013) and the Superscript III enzyme (ThermoFisher 18080085). cDNA was then diluted 1:5 after completion of reverse transcription. Fast SYBR Green Master Mix (Thermo Fisher 4385612) reagents were used to perform a ΔΔCt comparison of expression of gene-specific primers following hormone stripping with CSS, stimulation with E2, or stimulation with R1881 using the QuantStudio 6 Flex Real Time qPCR System. Primers for *AQP3 (F: 5’* CCGTGACCTTTGCCATGTGCTT 3’, R: 5’ TTGTCGGCGAAGTGCCAGATTG 3’*), SEC14L2 (F: 5’* CCTGAAGACCAAGATGGGAGAG 3’, R: 5’ GCTGTAGGTGTTGTCAAACCGC 3’)*, PGR* (F: 5’ AGGTCTACCCGCCCTATCTC 3’, R: 5’ AGTAGTTGTGCTGCCCTTCC 3’)*, AR (*F: 5’ CAGTGGATGGGCTGAAAAAT 3’, R: 5’ GGAGCTTGGTGAGCTGGTAG 3’)*, GREB1* (F: 5’ CAAAGAATAACCTGTTGGCCCTGC 3’, R: 5’ GACATGCCTGCGCTCTCATACTTA 3’), and *GAPDH* (F: 5’ TGCACCACCAACTGCTTAGC 3’, R: 5’ GGCATGGACTGTGGTCATGAG 3’) were obtained from Integrated DNA Technologies. The ΔΔCt comparison was calculated by comparing the genes of interest with GAPDH as an internal control, then comparing each condition to cells cultured in FBS as a control. The relative expression of each gene was then assessed. All experiments used three technical replicates, and data are represented as relative expression ± SEM from three independent experiments (*n* = 3).

### Drug information

Enzalutamide (MDV3100; HY-70002), apalutamide (ARN-509; HY-16060), darolutamide (ODM-201; HY-16985), tamoxifen (HY-13757A), fulvestrant (HY-13636) and β-estradiol (E2, HY-B0141) were obtained from MedChemExpress. Seviteronel (VT-464) was obtained from Innocrin Pharmaceuticals. ARD-61 was provided by the labs of Shaomeng Wang and Arul Chinnaiyan at the University of Michigan. R1881 was provided by the lab of Arul Chinnaiyan at the University of Michigan.

### Irradiation

A Kimtron IC-225 orthovoltage machine was used to provide X-ray radiation at the University of Michigan Experimental Irradiation Core. In keeping with previous work, a dose rate of 2 Gy/min was used [22, 29–31].

### Statistical analyses

GraphPad Prism 8.0 was used to perform all statistical analyses. For the determination of IC50s, a dose-response curve was calculated. For all in vitro experiments, a one-way ANOVA with Dunnett’s multiple comparisons or a one-way Student’s *t*-test was used to compare treatment groups. All data meet the assumptions for the statistical tests that were used, and the variance between comparison groups is similar.

## Results

### Validation of AR+/ER+ breast cancer cell lines in vitro

First, functional AR and ER activity was validated by western blot and through qPCR experiments in AR+/ER+ breast cancer cell lines (CAMA-1, ZR-75-1, BT-474; Supplementary Fig. 1A). To do this, nuclear fractionation experiments were performed to assess the cellular localisation of AR and ER under hormone depletion (CSS) or stimulated conditions (E2 or R1881). While β-estradiol or R1881 stimulation was sufficient to induce nuclear translocation of ERα or AR, respectively, there was little cross-reactivity between the hormone receptors, further suggesting that estrogen or androgen stimulation is specific for the activation of each receptor (Supplementary Fig. 1C–E), and R1881 does not cross stimulate ERα nuclear translocation or vice versa.

To functionally evaluate AR and ER activity in vitro, we performed RT-qPCR experiments to assess the transcription of ERα (*GREB1, PGR*) and AR (*SEC14L2, AQP3, AR*) target genes in CAMA-1, ZR-75-1 and BT-474 cells after stimulation of β-estradiol or R1881. First, *AR* transcript levels were assessed following CSS conditions or under stimulated conditions. While growth in CSS media was sufficient to induce an increase in levels of *AR* transcripts, stimulation with β-estradiol or R1881 resulted in a decrease in relative expression in CAMA-1 and BT-474 cells (Supplementary Fig. 2A, K). Treatment with β-estradiol in ZR-75-1 cells, however, increased the levels of *AR* transcript (Supplementary Fig. 2F). While R1881 stimulation was sufficient to significantly induce expression of *AQP3* in all three AR+/ER+ models (Supplementary Fig. 2B, G, L), expression of the AR target, *SEC14L2*, was induced in response to R1881 stimulation only in CAMA-1 and BT-474 (Supplementary Fig. 2C, M) but not ZR-75-1 cells (Supplementary Fig. 2H). Stimulation with β-estradiol was sufficient to induce transcription of ERα target genes (*GREB1, PGR*) in CAMA-1, BT-474 and ZR-75-1 cells (Supplementary Fig. 2D, E, I, J, N, O). Together, these findings suggest that, while functional in each AR+/ER+ in vitro model, AR may be playing a distinct role in ZR-75-1 cells compared to BT-474 and CAMA-1 due to the differences in *SEC14L2* and *AR* expression in response to androgen or estrogen stimulation.

### AR inhibition with second-generation anti-androgens does not radiosensitise AR+/ER+ breast cancer cell lines in vitro

**Fig. 1:**
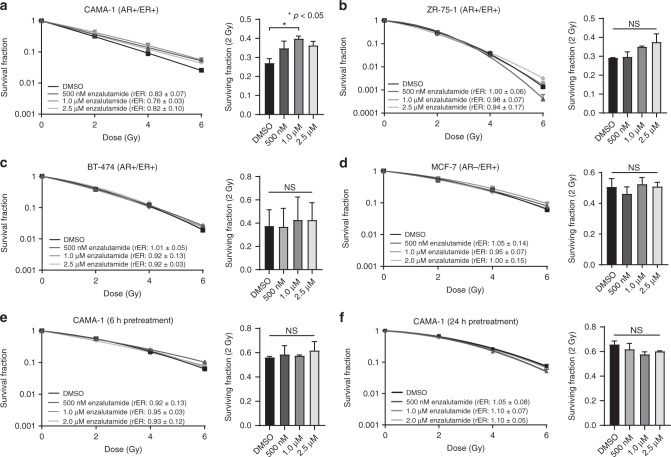
AR inhibition with enzalutamide does not affect radiosensitivity of AR+/ER+ breast cancer cell lines in vitro. Clonogenic survival assays were performed in AR+/ER+ **a** CAMA-1, **b** ZR-75-1 and **c** BT-474 cells to assess radiosensitisation with a 1 h pretreatment of enzalutamide prior to radiation treatment. Assays were also performed in **d** MCF-7 cells which are ER+ with low AR expression. To assess the effects of a longer pretreatment with enzalutamide, clonogenic survival assays were performed in CAMA-1 cells with a **e** 6 h or **f** 24 h pretreatment with enzalutamide prior to radiation treatment. Representative clonogenic survival assays are shown for each cell line, and the surviving fraction of cells at 2 Gy (SF-2Gy) are representative of three independent experiments (mean ± SEM). *p < 0.05; NS not significant.

To determine whether AR is acting independently from ER signaling in ER+ breast cancers to promote radioresistance, clonogenic survival assays were performed with the second-generation AR antagonist, enzalutamide. To do this, AR+/ER+ cells were treated with DMSO or 500 nM–2.5 µM enzalutamide for 1 h prior to radiation treatment. Enzalutamide treatment in combination with RT in AR+/ER+ CAMA-1 cells resulted in a significant increase in the surviving fraction of cells at 2 Gy (SF-2Gy; Fig. 1a). CAMA-1 cells treated with enzalutamide and RT had radiation enhancement ratios (rER) of 0.76–0.83 (Fig. 1a). In comparison, well-characterised radiosensitisation agents, like cisplatin, provide rER of 1.2 [32, 33], while drugs used for radioprotection, like amifostine, have rER of 0.8 [34]. In additional models, compared to treatment with DMSO, 0.5–2.5 µM enzalutamide had little effect on the radiosensitivity of ZR-75-1 cells (rER: 0.94–1.00, Fig. 1b) or BT-474 cells (rER: 0.92–1.01, Fig. 1c) with no change in SF-2Gy in either cell line. MCF-7 cells with high-ER expression but low AR expression (AR−/ER+) were also treated with 0.5–2.0 µM enzalutamide as a control. Enzalutamide had no effect on the radiosensitivity of MCF-7 cells (rER: 0.95–1.05) with no change in SF-2Gy with treatment (Fig. 1d). Therefore, enzalutamide treatment, at best, appears to have little effect on the radiosensitivity of AR+/ER+ breast cancer cells, and at worst, enzalutamide may protect AR+/ER+ cells from RT-induced cell death.

To assess the effect of variable drug pretreatment time on cell survival, AR+/ER+ CAMA-1 cells were treated with enzalutamide (0.5–2.0 µM) at 6 and 24 h prior to RT, in contrast to the 1 h pretreatment used in Fig. 1a–d. Increased pretreatment time had no effect on the observed radiosensitisation with rER of 0.92–0.95 for 6 h enzalutamide pretreatment (Fig. 1e) and rER of 1.05–1.10 when cells were pretreated for 24-h prior to RT (Fig. 1f). These data suggest that the longer pretreatment times are similarly insufficient at inducing radiosensitisation in AR+/ER+ breast cancer cells, and prolonged AR inhibition does not affect radiosensitivity despite its known role in inducing G1 cell cycle arrest [35, 36].

Next to further assess radiosensitivity using additional pharmacologic AR inhibitors, clonogenic survival assays were performed with apalutamide, darolutamide or seviteronel. While apalutamide and darolutamide are both second-generation AR antagonists, apalutamide is structurally similar to enzalutamide. In contrast, darolutamide is structurally unique compared to enzalutamide and apalutamide and has less blood-brain barrier penetration, offering a unique clinical application as serum testosterone levels are not altered in CRPC patients with darolutamide treatment [13, 37]. Seviteronel, a dual CYP17 lyase and AR inhibitor with some antagonist activity against ER [38], has been shown to be effective in AR+ TNBC models [39]. Similarly, the use of apalutamide is effective as a radiosensitisation strategy in AR+/ER− ACC-422 cells (rER: 1.08–1.33, Supplementary Fig. 1B). Using a 1 h pretreatment with apalutamide, darolutamide or seviteronel, we further assessed radiosensitisation in AR+/ER+ breast cancer cells. CAMA-1 cells had no change in radiosensitivity with 0.5–2.0 µM apalutamide (rER: 0.95–1.01, Supplementary Fig. 3A), or 0.5–2.0 µM darolutamide (rER: 0.92–0.93, Supplementary Fig. 3E). Similarly, ZR-75-1 cells treated with 0.5–2.0 µM apalutamide (rER: 0.93–1.04, Supplementary Fig. 3B), 0.5–2.0 µM darolutamide (rER: 0.92–1.1, Supplementary Fig. 3F) or 0.5–2.5 µM seviteronel (rER: 0.97–1.08, Supplementary Fig. 3I) had no change in radiosensitisation or SF 2 Gy. BT-474 cells also had no change in radiosensitisation with 0.5–2.0 µM apalutamide (rER: 0.96–0.98, Supplementary Fig. 3C), 0.5–2.0 µM darolutamide (rER: 0.94–0.97, Supplementary Fig. 3G) or 0.5–2.5 µM seviteronel (rER: 0.90–0.98, Supplementary Fig. 3J), further suggesting that though these inhibitors have structural and potential functional differences, each is insufficient under these conditions to induce radiosensitisation of AR+/ER+ breast cancer cell lines in vitro.

As a control, clonogenic survival assays were also performed in AR−/ER+ MCF-7 cells, where treatment with 0.5–2.0 µM apalutamide (rER: 0.85–0.98, Supplementary Fig. 3D), or 0.5–2.0 µM darolutamide (rER: 1.02–1.10, Supplementary Fig. 3H) did not alter radiosensitisation. In contrast to findings in AR+ TNBC [8], together these results suggest that 1 h pretreatment of apalutamide, or darolutamide, like enzalutamide, does not result in radiosensitisation of AR+/ER+ breast cancer cells in vitro. Further, our results suggest that methods of targeting AR signaling using pharmacologic AR antagonists and/or blocking androgen production with CYP17 inhibitors are insufficient to alter the radiation response in vitro in AR+/ER+ breast cancer models.

### PROTAC-mediated AR degradation in AR+/ER+ breast cancer cells

**Fig. 2:**
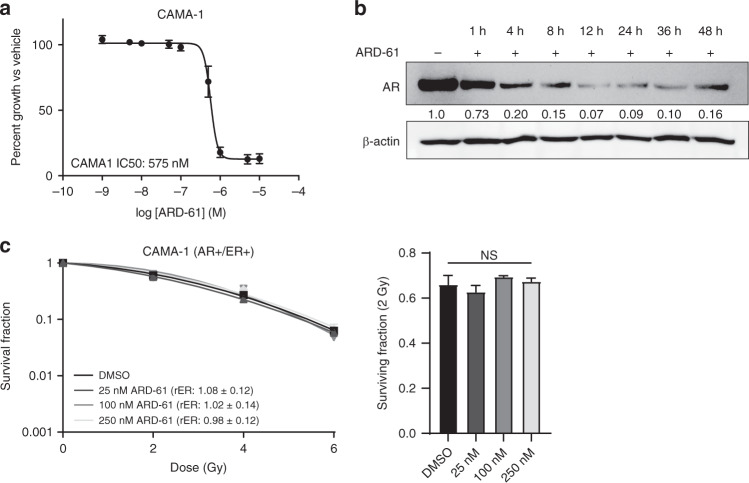
AR degradation with ARD-61 decreases cell viability but does not sensitise CAMA-1 cells to ionising radiation. **a** Cellular viability of AR+/ER+ CAMA-1 cells was assessed at 72 h with an IC50 of 575 nM. **b** Degradation of AR protein levels was assessed by western blot after 1–48 h treatment of ARD-61 with maximal degradation of AR occurring between 12 and 36 h. **c** Radiosensitisation of CAMA-1 cells with ARD-61 was assessed by clonogenic survival assay. Viability assays are the shown as the mean ± SEM for three independent experiments. A representative western blot is shown with quantification of average AR protein from three independent experiments. A representative clonogenic survival assay is shown with the SF-2Gy from three independent experiments (mean ± SEM). NS not significant.

Multiple studies have demonstrated that pharmacologic inhibition has different effects compared to protein depletion through genetic knockdown or the use of pharmacologic degraders [40, 41]. Having observed no change in radiosensitisation with pharmacologic inhibition of AR, we used novel proteolysis targeting chimera (PROTAC) AR degrader, ARD-61, to assess radiosensitisation [42, 43]. ARD-61 was effective at inhibiting cell viability in CAMA-1 cells with an IC50 of 575 nM (Fig. 2a). The kinetics of AR degradation in CAMA-1 cells was assessed by western blot and optimal degradation was found to occur from 12–36 h post-ARD-61 treatment where ≥90% of AR protein is degraded with 250 nM treatment of ARD-61 (Fig. 2b). Next, clonogenic survival assays were performed with 25–250 nM ARD-61 in AR+/ER+ breast cancer cells. Twenty-four hours pretreatment with ARD-61 was used to achieve maximal AR degradation prior to RT. ARD-61-mediated AR degradation did not change radiosensitisation of CAMA-1 cells with rER of 0.98–1.08 and no change in SF-2Gy (Fig. 2c), suggesting that pharmacologic degradation of AR has a similar lack of effect on radiosensitisation compared to pharmacologic inhibition in AR+/ER+ CAMA-1 cells.

### Dual inhibition of AR and ER together does not radiosensitise AR+/ER+ cell lines

**Fig. 3:**
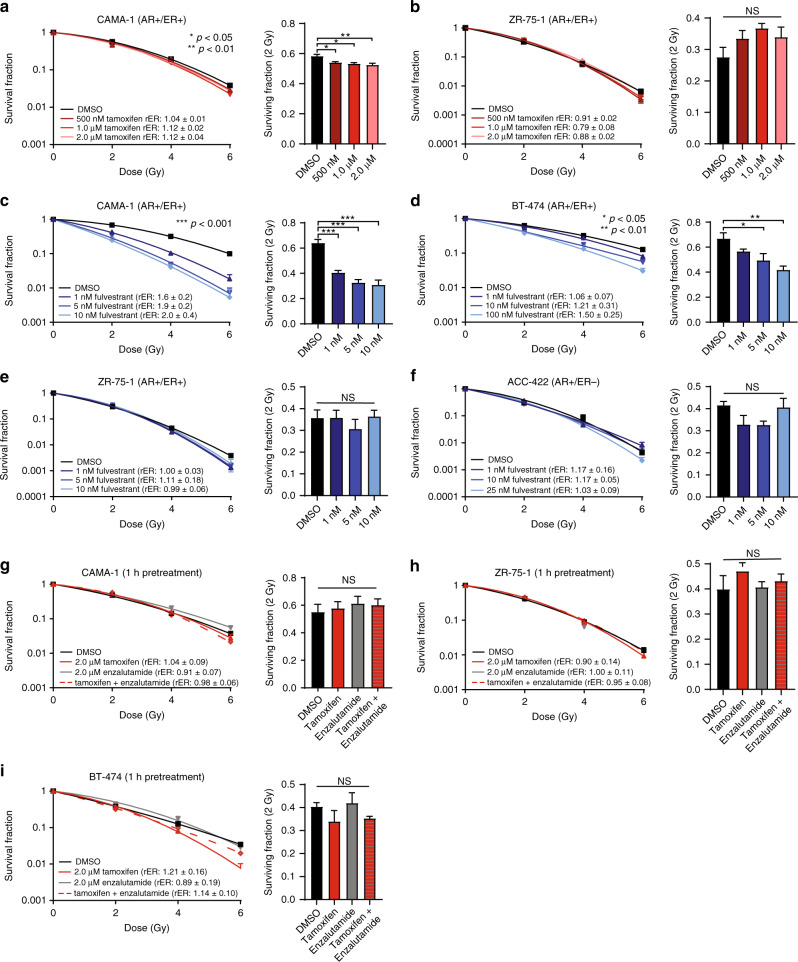
ER inhibition ± AR inhibition with enzalutamide is not sufficient to radiosensitise AR+/ER+ breast cancer cells in vitro. Clonogenic survival assays were performed in **a** CAMA-1 or **b** ZR-75-1 cells with a 1 h pretreatment of tamoxifen prior to radiation treatment. Radiosensitisation was assessed by clonogenic survival assays in **c** CAMA-1, **d** BT-474 and **e** ZR-75-1 cells with treatment of fulvestrant, a known degrader of both ER and AR protein. **f** Clonogenic survival assays were also performed in ACC-422 cells (AR+/ER−) with fulvestrant. Additional clonogenic survival assays were performed in **g** CAMA-1, **h** ZR-75-1 or **i** BT-474 cells with a 1 h pretreatment of enzalutamide, tamoxifen, or enzalutamide and tamoxifen. Representative clonogenic survival assays are shown for each cell line, and the SF-2Gy are representative of three independent experiments (mean ± SEM). *p < 0.05; **p < 0.01; ***p < 0.001; NS not significant.

To understand whether there were overlapping roles for AR and ER in AR+/ER+ breast cancer models, clonogenic survival assays were performed with the selective estrogen receptor modulator (SERM), tamoxifen, or the selective estrogen receptor degrader (SERD), fulvestrant. While previous work has demonstrated a role for tamoxifen or fulvestrant in the radiosensitisation of ER+ breast cancer models in vitro and in vivo [22], the role of AR and ER together and the impact on radiosensitisation has not been assessed. ER inhibition with 0.5–2.0 µM tamoxifen treatment alone in CAMA-1 cells resulted in only slight increases in radiosensitisation with rER of 1.04–1.12 (Fig. 3a). In ZR-75-1 cells, 0.5–2.0 µM tamoxifen treatment resulted in slight radioprotection with rER of 0.79–0.91 (Fig. 3b). These results suggest that inhibition of ER with tamoxifen was insufficient to alter radiosensitivity in AR+/ER+ breast cancer models in vitro. CAMA-1 cells were also pretreated with 1–10 nM fulvestrant for 1 h before RT. Fulvestrant, unlike tamoxifen, did radiosensitise CAMA-1 (rER: 1.6–2.0, Fig. 3c) and BT-474 (1–100 nM, rER: 1.06–1.50, Fig. 3d) cells but not AR+/ER+ ZR-75-1 cells (1–10 nM, rER: 0.99–1.11, Fig. 3e) or AR+/ER− ACC-422 cells (1–25 nM, rER: 1.03–1.17, Fig. 3f). These data indicate that ER degradation with short-term fulvestrant may be sufficient to sensitise select AR+/ER+ cell lines to ionising radiation but is not sufficient to radiosensitise all AR+ breast cancer cell lines.

We next wanted to understand whether AR and ER may have an overlapping function allowing the hormone receptors to compensate in response to radiation treatment. To investigate this, clonogenic survival assays were performed in CAMA-1, ZR-75-1 and BT-474 cells pretreated for 1 h with 2.0 µM tamoxifen and/or 2.0 µM enzalutamide prior to RT. There was no significant change in radiosensitisation in cells treated with tamoxifen alone (rER: 0.90–1.21), enzalutamide alone (rER: 0.89–1.00) or the combination of tamoxifen and enzalutamide (rER: 0.95–1.14, Fig. 3g–i). These data suggest that short-term pretreatment with both ER and AR inhibitors is not sufficient to block a potential compensatory mechanism between AR and ER in response to ionising radiation.

To determine whether extended pretreatment of AR+/ER+ cells with tamoxifen and/or enzalutamide treatment resulted in changes in radiosensitivity, cells were treated with the drug once daily for seven days prior to RT. While ZR-75-1 cells had little change in radiosensitisation with this extended pretreatment (rER with tamoxifen: 1.00 ± 0.24, rER with enzalutamide: 1.16 ± 0.3, rER with tamoxifen and enzalutamide: 1.10 ± 0.07, Supplementary Fig. 4A), CAMA-1 cells showed slight radiosensitisation with tamoxifen alone (rER: 1.26 ± 0.11) as well as with the combination treatment (rER: 1.18 ± 0.03, Supplementary Fig. 4B). There was also a decrease in the SF-2Gy of CAMA-1 cells with this extended duration of treatment prior to RT. This effect could be due to epigenetic changes that take place over the prolonged exposure to tamoxifen [44]. Together these results suggest that antagonising both AR and ER together is insufficient to radiosensitise AR+/ER+ breast cancer cells in vitro further suggesting that while AR and ER may have overlapping functions, the receptors may not be working together to mediate radioresistance in models of breast cancer where they are co-expressed.

### AR knockout in combination with ER antagonists does not radiosensitise AR+/ER+ cells

**Fig. 4:**
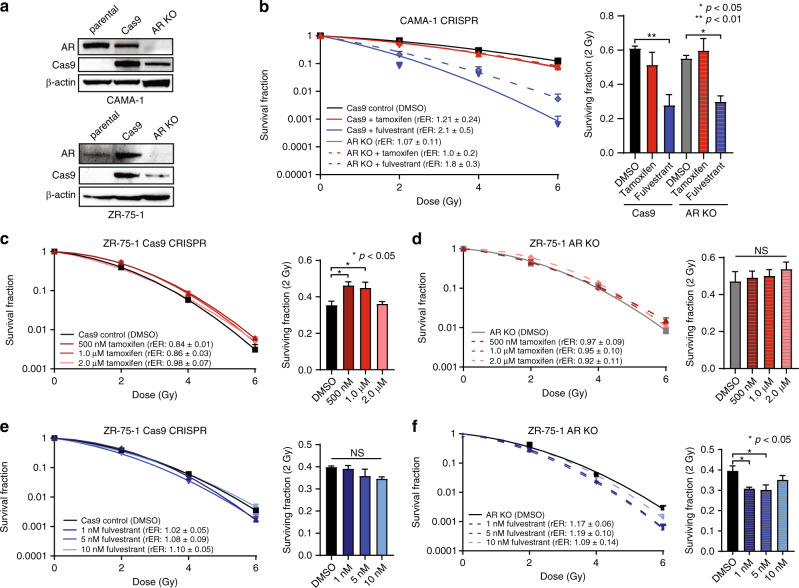
Knockout of AR by CRISPR/Cas9 is not sufficient to provide radiosensitisation to AR+/ER+ cells alone or in combination with ER inhibitors. CRISPR/Cas9-mediated knockout of *AR* was confirmed by western blot **a** in CAMA-1 and ZR-75-1 cells. **b** Radiosensitisation of CAMA-1 AR KO or Cas9 control cells was assessed by clonogenic survival assay alone or in combination with tamoxifen or fulvestrant treatment. Radiosensitisation of ZR-75-1 **c** Cas9 control cells or **d** AR KO cells with tamoxifen was assessed by clonogenic survival assay. Similarly, radiosensitisation with fulvestrant was assessed in ZR-75-1 **e** Cas9 control or **f** AR KO cells. Representative clonogenic survival assays are shown for each cell line, and the SF-2Gy are representative of three independent experiments (mean ± SEM). *p < 0.05, **p < 0.01; NS not significant.

Having observed no radiosensitisation when AR and ER were pharmacologically inhibited, we next tested whether genomic deletion of *AR* using CRISPR-Cas9 conferred radiosensitisation. These experiments were used to further investigate the overlapping roles of AR and ER in these models. Isogenic cell line models with *AR* knockout (AR KO) or Cas9 control cells containing the Cas9 protein and a control guide (*AAVS1*) were generated in CAMA-1 or ZR-75-1 (Fig. 4a) cells which endogenously express both AR and ER. Clonogenic survival assays were then performed using CAMA-1 and ZR-75-1 AR KO or Cas9 cells. To investigate whether ER antagonism was sufficient to radiosensitise CAMA-1 cells with genetic AR KO, CAMA-1 AR KO and Cas9 control cells were treated with tamoxifen or fulvestrant. In support of results observed with pharmacologic inhibition of AR in these cell lines, there was no change in radiosensitisation in CAMA-1 Cas9 control cells compared to AR KO cells (Fig. 4b). Treatment of Cas9 control cells with 2.0 μM tamoxifen, however, provided slight radiosensitisation (rER: 1.21 ± 0.24). As observed in CAMA-1 parental cells, treatment of Cas9 control cells with 10 nM fulvestrant increased radiosensitivity (rER: 2.1 ± 0.5). In CAMA-1 AR KO cells, however, there were similar levels of radiosensitisation with tamoxifen (rER: 1.00 ± 0.2) and fulvestrant (rER: 1.8 ± 0.3, Fig. 4b) when compared to Cas9 control cells under similar treatment conditions. These results suggest that the presence or absence of AR in combination with treatment of tamoxifen or fulvestrant does not alter the observed radiosensitisation. Radiosensitisation of AR KO cells with ER antagonists was similar to observed radiosensitisation in Cas9 cells further suggesting that genetic knockout of AR in combination with ER inhibition was not sufficient to sensitise AR+/ER+ cells to ionising radiation. There is no radiosensitisation synergy observed between tamoxifen or fulvestrant treatment in CAMA-1 AR KO cells compared to Cas9 control.

Similar results were observed in ZR-75-1 AR KO or Cas9 control cells treated with tamoxifen or fulvestrant. In ZR-75-1 Cas9 control cells, no significant effects were observed as a result of treatment with 0.5–2.0 μM tamoxifen (rER: 0.84–0.98, Fig. 4c). This result was mirrored in ZR-75-1 AR KO cells with tamoxifen treatment (rER: 0.92–0.97, Fig. 4d). Similar to results seen in ZR-75-1 parental cells, treating ZR-75-1 Cas9 control cells with fulvestrant did not result in significant radiosensitisation with enhancement ratios of 1.02–1.10 (Fig. 4e). In the same way, ZR-75-1 AR KO cells had enhancement ratios of 1.09–1.19 with 1–10 nM fulvestrant (Fig. 4f). Together these results suggest that genetic knockout of AR protein in combination with ER inhibition or degradation is insufficient to radiosensitise AR+/ER+ CAMA-1 or ZR-75-1 cells, although there are cell-specific differences in radiosensitisation with tamoxifen or fulvestrant, respectively. Although AR inhibition in TNBC is radiosensitising [7, 8], as is ER inhibition in ER+ breast cancers with low AR expression [22], these findings further suggest that AR and ER signaling are not converging on a single pathway affecting the radiation response when AR and ER are co-expressed in models of breast cancer, or that there may be additional factors involved in the radiation response in AR+/ER+ breast cancer models.

## Discussion

While previous studies have demonstrated that AR inhibitors can radiosensitise AR+ models of TNBC, prostate cancer and glioblastoma [7, 8, 16, 25, 27, 45, 46], in this study we demonstrate that pharmacologic inhibition, degradation, or genetic knockout of AR using CRISPR-Cas9, is not sufficient to radiosensitise AR+/ER+ breast cancer cell lines in vitro (Figs. 1, 2, 4, Supplementary Fig. 3). While effective in ER+ MCF-7 and T47D cells [22], 1 h pretreatment with the ER inhibitors tamoxifen or fulvestrant results in minimal radiosensitisation of AR+/ER+ cell lines (Fig. 3). Further, the combination treatment of AR and ER inhibition via tamoxifen with enzalutamide is not sufficient to radiosensitise AR+/ER+ cells in vitro (Fig. 3, Supplementary Fig. 4). Knockout of *AR* using CRISPR-Cas9 does not affect cell radiosensitivity relative to Cas9 control cells, and the addition of tamoxifen did not provide synergistic radiosensitisation effects in CAMA-1 or ZR-75-1 cells (Fig. 4). Conversely, treatment with fulvestrant, a selective estrogen receptor degrader (SERD) with some ability to degrade AR protein [47], was sufficient to radiosensitise CAMA-1 parental or Cas9 control cells; however, similar levels of radiosensitisation were observed in CAMA-1 AR KO cells with fulvestrant treatment suggesting there is no additional synergistic effect of AR KO in combination with ER inhibition or degradation (Fig. 4). Together these findings suggest that AR inhibition alone is not sufficient to radiosensitise AR+/ER+ breast cancer models. In addition, combined abrogation of AR and ER pharmacologically or through gene editing does not provide radiosensitisation suggesting that AR and ER are not directly compensating for the radiation response.

Our findings here suggest an independent role for AR in AR+/ER+ breast cancer models in response to radiation compared to the previously established role for AR in the radiation response in AR+/ER− (TNBC) models and other AR+ cancers [15]. In AR+/ER− breast cancer, AR is a biomarker of radioresistance and inhibition or knockdown of AR results in radiosensitisation, partially through the inhibition of a non-homologous end-joining (NHEJ) repair-mediated response [7, 8]. Here, we demonstrate that in the presence of ER, AR may not be functioning as a mediator of radioresistance, suggesting that AR inhibition is not an effective radiosensitisation strategy in women with tumours expressing high levels of AR and ER. This is in contrast with previous work suggesting that AR inhibition, independent of ER status, results in radiosensitisation in AR+ breast cancer models [16]. This conflict may also be due to differences in treatment conditions and the timing of treatment. For example, it should be noted that other studies used proliferation assays instead of clonogenic survival assays to assess radiation response, and much higher doses of enzalutamide (10-fold higher) and radiation (10 Gy) were used in these studies which may explain these differences in findings.

Further, our data indicate that anti-androgen or anti-estrogen therapies may be effective radiosensitisation strategies in some, but not all AR+/ER+ breast tumours, suggesting a need for additional biomarkers of response for AR and/or ER antagonists. While an AR:ER positivity ratio of 78% has been demonstrated to have the prognostic capacity in AR+/ER+ tumours [48], a more complete understanding of how signaling of androgen and estrogen receptors, along with signaling of other receptors and growth factors, is influencing the radiation response is needed to effectively identify reliable biomarkers of response. Our work also suggests that the use of anti-estrogen therapies may be an effective radiosensitisation strategy in some, but not all, ER+ breast cancer models [22]. Notably, while our study employs multiple AR+/ER+ cell lines, these cell lines have different molecular characteristics, including levels of AR and ER protein expression and functional downstream signaling as assessed by RT-qPCR (Supplementary Figs. 1 and 2). These molecular differences may also contribute to the differences observed in response to pharmacologic AR and ER inhibitors in combination with RT. While in this study we demonstrate that AR expression may be influencing the efficacy of ER inhibitors in modulating radiosensitisation in AR+/ER+ breast cancer cells, future studies are necessary to understand why ER-targeting therapies may be effective for the radiosensitisation in some preclinical ER+ breast cancer models but not others. This may be due to the expression of additional biomarkers that can impact radiosensitivity including HER2 and EGFR [49–52], PI3K/mTOR [53, 54] and PARP [55–57], among others. While preclinical studies are useful to start interrogating these questions using models that have been studied extensively, translation of these findings into the clinic brings additional variabilities including, but not limited to, individual patient characteristics, adherence to treatment, as well as other concurrent therapies. Therefore, biomarkers of response are also needed to understand how AR agonists and antagonists might be used in different patient populations in diverse biological contexts to increase tumour control.

Despite these findings, there are also a few limitations to the current study that are worth noting. These studies use tamoxifen instead of 4-hydroxytamoxifen (4-OHT), which is an active metabolite of tamoxifen, and 100× more potent than tamoxifen [58]. As tamoxifen is a weaker anti-estrogen than 4-OHT and a partial agonist of ER [59, 60], the concentrations used may not be sufficient to induce radiosensitisation in media containing FBS with supersaturated E2 levels. Future experiments will seek to expand these findings and investigate whether a more potent inhibitor like 4-OHT may be a more effective radiosensitisation strategy in these models. Additionally, because all cell lines used in this study were cultured in media containing both phenol-red and FBS, we cannot control for exogenous androgens and estrogens that exist within the media [61]. While seviteronel is both a CYP17 and AR inhibitor, CYP17 is not commonly expressed in breast cancer cell lines, and the culture media used has supplemented cholesterol and steroid hormones, thus preventing cells from relying on CYP17 for androgen production. Together these limitations could also suggest that the use of anti-androgens may be more effective when assessed in models containing more physiologically relevant levels of circulating hormones. Future studies performed in xenograft models or using patient data from completed clinical trials should be performed to understand whether changes in estradiol and/or testosterone levels affect radiosensitivity with anti-androgen or anti-estrogen therapies. Finally, non-canonical ER functions including RNA binding [62] or non-genomic signaling [63] that may contribute to radiation response in the context of AR signaling cannot be excluded based on the data presented here and are also worth further exploration. Here we have investigated the role of AR and ER signaling using in vitro and immunocompromised models that eliminate any potential effects of the tumour microenvironment or immune infiltrates [64, 65]. Future studies may investigate these limitations by using immunocompetent in vivo models.

While AR-targeting therapies have become a mainstay in the treatment of prostate cancers, ongoing preclinical and clinical studies in AR+ tumours have demonstrated therapeutic potential for the treatment of AR+ breast cancers using AR antagonists. In a phase II trial (NCT01889238), enzalutamide has been shown to provide clinical benefit to ~30% of AR+ TNBC patients [6]. Additionally, a phase I trial is underway using darolutamide in breast cancer patients, including TNBC, HR+/HER2− and HER2+ patients (NCT03004534). Recent data have also suggested that AR functions as a tumour suppressor in AR+/ER+ breast cancers [17], and ongoing trials are investigating the use of AR agonists (NCT01616758, NCT02463032). In addition, there are many ongoing clinical trials are investigating the concurrent use of anti-androgen and anti-estrogen therapies (including NCT02953860, NCT02955394, NCT02676986). The results from these trials will continue to shed light on the use of antagonists and agonists for the treatment of AR+ and/or ER+ breast tumours. Further, our work highlights the importance of understanding how AR and ER interact to influence tumorigenesis to appropriately direct clinical trial design and stratify patient populations most effectively, as well as to inform the timing of radiation treatment in relation to AR inhibition as these studies suggest co-treatment may be tumour protective and thus undesirable.

## Supplementary information

Supplementary Figures and Captions

AJ Checklist
