# Supplementary material for: Androgen and oestrogen receptor co-expression determines the efficacy of hormone receptor-mediated radiosensitisation in breast cancer
Source: Br J Cancer. 2022 May 26;127(5):927–36. doi: 10.1038/s41416-022-01849-9 (PMC9427858; doi:10.1038/s41416-022-01849-9)
Supplement: Supplementary file 1 — Supplementary Figures and Captions [file 41416_2022_1849_MOESM1_ESM.docx]

**Supplementary Figure 1: Validation of AR function in AR+ breast cancer models.** Expression of AR and ERα was assessed by western blot (**A**) in a panel of breast cancer cell lines. Clonogenic survival assays were performed in (**B**) AR+/ER- ACC-422 cells treated with apalutamide for one hour prior to RT to assess radiosensitization. Western blots were performed in AR+/ER+ (**C**) CAMA-1, (**D**) ZR-75-1, and (**E**) BT-474 cells to assess cellular localization (nucleus or cytosol) for AR or ERα following hormone depletion (CSS) or stimulation with 1 nM β-estradiol (E2) or R1881. A representative clonogenic survival assay is shown for the ACC-422 cells, and the SF-2Gy values are representative of three independent experiments (mean ± SEM). Nuclear fractionation experiments were performed in duplicate or triplicate, and a representative blot is shown. ** p < 0.01

**Supplementary Figure 2: Expression of AR and ER target genes was assessed in AR+/ER+ breast cancer cell lines.** qPCR experiments were performed to assess expression of AR target genes (*AR, AQP3, SEC14L2*) and ER target genes (*GREB1,* *PGR*) in AR+/ER+ (**A-E**) CAMA-1, (**F-J**) ZR-75-1, or (**K-O**) BT-474 cells grown in FBS or CSS, stimulated ± 1 nM β-estradiol or R1881. Data is shown as mean ± SEM for three independent experiments. * p < 0.05; ** p < 0.01; *** p < 0.001; **** p < 0.0001; NS = not significant

**Supplementary Figure 3: Inhibition with apalutamide, darolutamide, or seviteronel does not affect radiosensitivity of AR+/ER+ breast cancer cell lines *in vitro*.** Clonogenic survival assays were performed in AR+/ER+ (**A**) CAMA-1, (**B**) ZR-75-1, and (**C**) BT-474 cells to assess radiosensitization with a one-hour pretreatment of apalutamide prior to radiation treatment. Assays were also performed in ER+ (**D**) MCF-7 cells with low AR expression. Similarly, clonogenic survival assays were performed in AR+/ER+ (**E**) CAMA-1, (**F**) ZR-75-1, and (**G**) BT-474 cells to assess radiosensitization with a one-hour pretreatment of darolutamide prior to radiation treatment. Assays were also performed in (**H**) MCF-7 cells. Next, clonogenic survival assays were performed in (**I**) ZR-75-1 and (**J**) BT-474 cells treated with seviteronel for one-hour prior to RT. Representative clonogenic survival assays are shown for each cell line, and the SF-2Gy are representative of three independent experiments (mean ± SEM). * p < 0.05; NS = not significant

**Supplementary Figure 4: Combined treatment of tamoxifen with enzalutamide does not radiosensitize AR+/ER+ breast cancer cell lines. (A)** Extended, one-week treatment with enzalutamide, tamoxifen, or enzalutamide and tamoxifen was delivered every 24 hours for 7 days prior to assessment of radiosensitization by clonogenic survival assays in **(A)** ZR-75-1 or **(B)** CAMA-1 cells. Representative clonogenic survival assays are shown for each cell line, and the SF-2Gy are representative of three independent experiments (mean ± SEM). * p < 0.05; ** p < 0.01; NS = not significant
